# Innate immunity in tumors: roles and therapeutic targets

**DOI:** 10.3389/fimmu.2025.1689714

**Published:** 2025-10-22

**Authors:** Songze Leng, Yuyue Ren, Yaoyao Tian, Weiwei Zhao, Yue Mou, Xingyu Chen, Hong Zhou, Wei Wang

**Affiliations:** ^1^ Department of Hematology, The Second Affiliated Hospital of Harbin Medical University, Harbin, Heilongjiang, China; ^2^ Harbin Medical University, Harbin, Heilongjiang, China

**Keywords:** innate immunity, tumor microenvironment, immunotherapy, immune cells, immune factors

## Abstract

Innate immune cells and pathways are central to shaping the tumor microenvironment (TME), where they influence tumor growth, metastasis, and responsiveness to immunotherapy. Although research on innate immunity in cancer has expanded considerably, the mechanisms driving immune dysfunction remain incompletely understood. This review summarizes current knowledge on the functional states of innate immune cells within the TME and highlights how metabolic reprogramming contributes to immune suppression and tumor progression. We further discuss recent advances in therapeutic strategies targeting innate immune pathways, emphasizing their translational potential. Importantly, we also examine unresolved controversies and knowledge gaps across innate immune cells, metabolic networks, and innate immune factors such as complement and cytokines, outlining key challenges for clinical translation. By linking mechanistic insights with emerging interventions and identifying future directions, this review provides a framework for integrating innate immunity into next-generation cancer treatment.

## 1 Introduction

**Figure 1:**
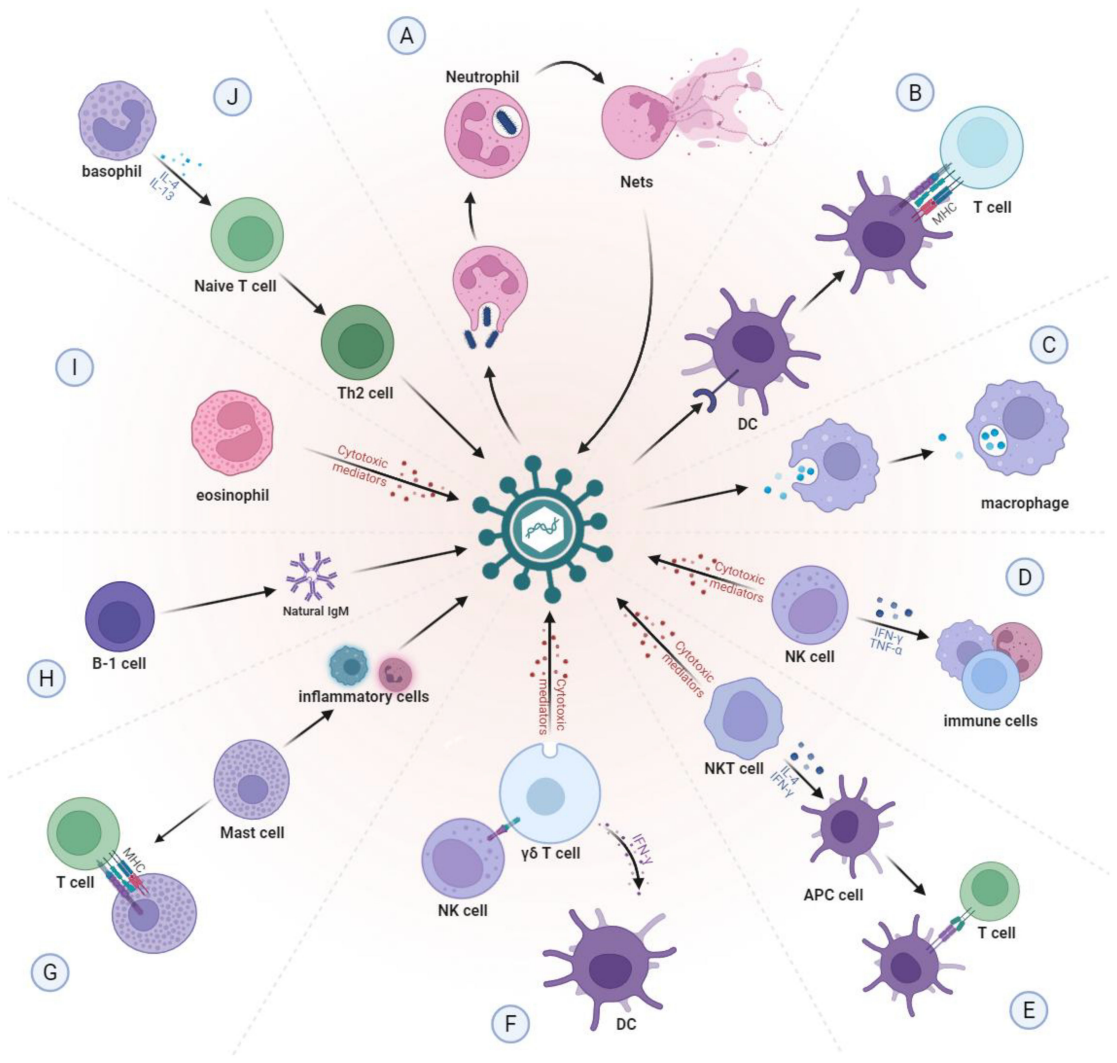
Normal function of innate immune cells. **(A)** Neutrophils can kill pathogens by directly engulfing pathogens or by self-sacrifice to create neutrophil extracellular traps (NETs). **(B)** DCs capture, process and present antigens to T cells through MHC molecules. **(C)** Macrophages phagocytose and remove foreign bodies and senescent cells from the body. **(D)** NK cells can quickly recognize and clear pathogens, while activating other immune cells by secreting cytokines. **(E)** NKT cells can directly kill pathogens and can also produce IFN-γ and IL-4 to activate APC cells. **(F)** γδ T cells can recognize and respond to a variety of antigens, act as APCs to present antigens to T cells, and activate other immune cells. **(G)** When pathogens invade, mast cells are stimulated and release various inflammatory mediators, which are able to attract more leukocytes to the site of infection. Mast cells also function as APCs. **(H)** B-1 cells can produce natural IgM antibodies, which can bind to a variety of pathogen associated carbohydrate antigens and play an early defense role. **(I)** Eosinophils mainly regulate type I hypersensitivity and kill foreign pathogens through cytotoxic effects. **(J)** Basophils can promote the activation of Th2 cells and enhance the humoral immune response by secreting cytokines. NETs, neutrophil extracellular traps; DCs, dendritic cells; MHC, major histocompatibility complex; NK, natural killer; NKT, natural killer T; IFN-γ, interferon-γ; IL, interleukin; APC, antigen-presenting cell; IgM, immunoglobulin M; Th2, T helper type 2.

Innate immunity represents the body’s intrinsic, non-specific defense mechanism and plays an equally critical role in tumor immune responses (**Figure 1**). Under ideal circumstances, abnormal cells would be promptly recognized and eliminated by the host immune system. However, as tumors progress, cancer cells acquire the ability to secrete diverse cytokines and chemokines that progressively impair immune cell function and foster immune evasion (1).

Immunotherapy has transformed the landscape of oncology, yet its clinical benefit remains restricted to a subset of patients. Current approaches predominantly focus on adaptive immunity—particularly T cell checkpoint blockade—while the crucial roles of innate immune cells and pathways have not been fully appreciated. Growing evidence now indicates that innate immunity not only initiates antitumor responses but also shapes the tumor microenvironment (TME) in ways that ultimately determine the effectiveness of adaptive immunity (2).

In this review, we provide a comprehensive overview of the innate immune system in cancer and analyze its therapeutic implications. We place particular emphasis on the intersection between innate immune metabolism and immunotherapy, and we highlight feasible strategies to harness innate immune pathways in the design of next-generation therapies. Importantly, we also discuss ongoing controversies, unresolved knowledge gaps, and future research directions, aiming to provide a balanced perspective that can inform the rational integration of innate immunity into personalized cancer treatment.

## 2 Innate immune cells in tumors

Innate immunity involves various bone marrow-derived cells, each contributing significantly to the innate immune response (3). As tumors develop, a corresponding TME is established, which is the internal milieu that nurtures tumor cells. Within the TME, tumor cells have the capacity to boost or suppress the innate immune response through the secretion of cytokines and chemokines (4). The interplay among various components of the TME significantly influences the functionality of innate immune cells. The following sections will further explore the functional alterations of innate immune cells within tumors and the underlying mechanisms that drive these changes (**Table 1**).

**Table 1:** Functional changes in innate immune cells in tumors.

Innate immune cells	Tumors	Functions and changes in tumors	References
Neutrophils	breast cancer, fibrosarcoma, colon cancer, promyelocytic leukemia	polarization of N1	(6, 7)
	lung cancer	polarization of N2	(8)
	lung cancer	promotion of tumor metastasis	(9)
	gastric cancer, lung cancer, colon cancer	promoting angiogenesis	(10, 11)
DCs	myeloma, melanoma	decreased ability to present antigen	(14, 15)
	prostate cancer	the migration ability of DC was weakened	(16)
	biliary tract cancer, colorectal cancer	inhibition of T cell activation	(18, 19)
Macrophages	colorectal cancer	shaping the immunosuppressive microenvironment	(22)
	oral squamous cell carcinoma, HCC	Promoting tumor cell proliferation	(21)
	non-small cell lung cancer, colon cancer	promoting tumor proliferation and invasive properties	(23, 29)
	hepatocellular carcinoma, gastric cancer, liver cancer.	promoting tumor immune escape	(24–26)
	osteosarcoma	improving the prognosis of patients	(28)
	non-small cell lung cancer	enhancing antitumor immunity	(30)
ILCs	acute myeloid leukemia, pancreatic cancer, glioblastoma, lung cancer	antitumor effects	(41)
	lung cancer, prostate cancer	reducing tumor growth	(42)
	colorectal cancer	preventing tumor progression	(43)
NK cells	HCC	killing tumor cells	(37)
	ovarian	the killing effect on tumor cells was inhibited	(36)
	liver cancer	recognition of reduced tumor function	(35)
NKT cells	HCC	proliferation is inhibited, and senescence occurs	(44)
	liver cancer	inhibition of tumors	(47)
	CLL, neuroblastoma	indicates a good prognosis	(45, 46)
	melanoma, neuroblastoma, lung cancer, colon cancer	regulating macrophages and inhibits tumor growth	(78)
	liver cancer	promotion of tumor metastasis	(48)
γδT cells	nonsmall-cell lung cancer	promoting tumor metastasis	(51)
	acute myeloid leukemia	acute myeloid leukemia	(50)
	colorectal cancer	promoting tumor immune escape and progression	(52)
B-1 cells	lymphoma, liver cancer, breast cancer	killing tumor cells	(55)
	melanoma	promotion of tumor metastasis	(57)
	B-CLL	promoting tumorigenesis	(56)
MCs	thyroid cancer, bladder cancer	promoting tumor progression	(58, 59)
Eosinophils	breast cancer	promoting lymphocyte-mediated antitumor immunity	(62)
	melanoma	promotion of tumor metastasis	(66)
	pancreatic cancer	inhibition of tumorigenesis	(63)
Basophils	CML	promoting tumor spread and development	(69)
	pancreatic ductal adenocarcinoma	tumor-promoting Th2 inflammation	(68)
	lung cancer	promoting tumor growth	(70)
MDSCs	Melanoma	promoting tumor angiogenesis, metastasis and microenvironment immunosuppression	(74)
	HCC	inhibit the infiltration and activity of T cells	(75)
	renal clear cell carcinoma, breast cancer	inhibit T cell function and promotes tumor progression	(76)
	breast cancer	promoting immunosuppressive function	(77)

### 2.1 Neutrophils

Neutrophils are the predominant cell type within the innate immune system and the first line of defense against infections. Within the TME, there is a significantly increase in neutrophil infiltration, accompanied by alterations in the function (5).

Although traditionally viewed as immunosuppressive, recent studies have highlighted their complex roles in tumors. In the early stages of tumor development, signaling molecules such as CXCL9 and IFN-β drive neutrophils toward an anti-tumor N1 phenotype. These cells are highly cytotoxic and enhance local immune responses by secreting pro-inflammatory chemokines (6, 7).

However, as tumors progress, cytokines such as IL-6, IL-10, TGF-β1, and G-CSF, neutrophils are polarized to the pro-tumor N2 phenotype, which exhibits immunosuppressive properties (8, 9). N2 neutrophils facilitate tumor angiogenesis by releasing factors such as vascular endothelial growth factor (VEGF) and matrix metalloproteinase-9 (MMP-9), and fibroblast growth factor-2 (FGF-2), ultimately promoting tumor proliferation and metastasis (10, 11). Additionally, neutrophils can form neutrophil extracellular traps (NETs) in response to specific stimuli. These web-like structures, while capable of trapping tumor cells and limiting their dissemination, also contribute to tumor progression by fostering an immunosuppressive microenvironment (12).

### 2.2 Dendritic cells

Antigen-presenting cells (APCs), primarily consisting of dendritic cells (DCs), play a crucial role in the innate immune system’s ability to recognize tumors. Widely distributed in nearly all tissues, DCs serve as a vital link between the innate and adaptive immune systems. They are responsible for capturing, processing, and presenting tumor antigens to naive T cells via major histocompatibility complex (MHC) molecules. MHC class I (MHC-I) presents endogenous antigens, while MHC class II (MCH-II) is responsible for handling exogenous antigens, thereby triggering adaptive immune responses characterized by CD8^+^ or CD4^+^ T cell activation (13).

The number of DCs is decreased in various tumors, correlating with tumor size and stage. These cells also show notable alterations in the phenotypic profiles. Specifically, reduced expression of HLA-A, B, C, DR, CCR5, CCR7 and DEC-205 in DCs compared to healthy controls (14). Among these, HLA-DR serves as a critical marker of DC maturation, while the other MHC molecules are essential for effective antigen presentation. CCR5 and CCR7, as chemokine receptors, are important for DC migration, and their downregulation further impairs DC trafficking and T cell priming within the tumor microenvironment (15–18). Moreover, abnormal upregulation of immunosuppressive signals critically contributes to DC dysfunction. Evidence indicates that lowering PD-L1 expression enhances CD8^+^ T cell anti-tumor activity and suppresses tumor growth (19).

### 2.3 Macrophages

Macrophages are essential components of the innate immune system, involved in various physiological processes such as pathogen clearance, tissue repair, and inflammation regulation. In tumor development, macrophage infiltration is a hallmark of solid tumors. Within the TME, macrophages are exposed to complex stimuli and exhibit high plasticity with diverse activation states. Traditionally, macrophages are categorized into two primary phenotypes: M1 and M2 (20). M1 macrophages are activated by pro-inflammatory factors such as IFN, CSF, and TNF. In contrast, M2 macrophages are activated by cytokines like TGF-β, IL-4, IL-13, and others.

Tumor-associated macrophages (TAMs) are highly heterogeneous and can either promote tumor progression or support anti-tumor immunity, depending on their functional states and the microenvironmental context. Beyond the classical M1/M2 dichotomy, multiple specialized TAM subsets have been identified. SPP1^+^ macrophages contribute to tumor metastasis, angiogenesis, and activation of cancer stem cells, and interact with FAP^+^ fibroblasts to form immuno-repulsive tissue structures that limit T cell infiltration (21–23). TREM2^+^ TAMs accumulate lipids via scavenger receptors, suppress CD8^+^ T cell activity, and enhance Treg-mediated immunosuppression (24). Additionally, inhibition of MARCO^+^ macrophages promotes the activation of CD8^+^ T cells and NK cells, thereby reprogramming the TME (25). In contrast, FOLR2^+^ macrophages positively correlate with CD8^+^ T cells and activate their cytotoxicity through antigen cross-presentation (26). Notably, C1q^+^ TAMs display a dual role. These cells can promote tumor progression by driving T cell exhaustion and correlating with poor prognosis, while also mediating anti-tumor immunity through proinflammatory and phagocytic functions that enhance therapeutic responses (27–29). Mechanistically, C1q^+^ TAMs interact with cancer stem cells via C1q–C1q receptor signaling, modulate cytokine secretion (e.g., IL-6, MCP-1) to either promote tumor progression or, through the AMPK/JAK/STAT pathway, enhance anti-tumor immunity (29, 30). Therapeutically, targeting TAMs can be achieved by inhibiting immunosuppressive subsets (e.g., C1Q^+^TPP1^+^), activating antitumor subsets (e.g., FABP4^+^C1q^+^), or combining with immune checkpoint blockade, highlighting macrophage-centered strategies as promising avenues for cancer immunotherapy (29–31). Collectively, these findings underscore the importance of recognizing TAM heterogeneity and leveraging specific subsets to optimize anti-tumor efficacy.

### 2.4 NK cells

Natural killer (NK) cells are a critical component of the innate immune system, capable of eliminating viral infections and certain tumor cells. In addition to their cytotoxic function, NK cells play essential roles in immune surveillance and regulation.

However, within the TME, NK cells often become functionally impaired, resulting in reduced anti-tumor activity (32). Tumor and stromal cells in the TME secrete various immunosuppressive factors, which directly or indirectly inhibit NK cell activation (33). These factors also suppress the activation of key NK cell receptors such as NKG2D and the tumor necrosis factor-related apoptosis-inducing ligand (TRAIL) (34). Additionally, NK cells in the TME frequently exhibit reduced membrane protrusions, a morphological alteration that may compromise their ability to recognize and eliminate tumor cells (35). Their proliferation, cytotoxicity, and granzyme B secretion are also significantly diminished under such conditions (36). Despite the immunosuppressive nature of the TME, NK cells in hepatocellular carcinoma (HCC) have been shown to retain strong cytotoxic activity, highlighting their potential as a target for cancer immunotherapy (37).

### 2.5 ILCs

Innate lymphoid cells (ILCs) are key components of the innate immune system and include three major subsets—ILC1, ILC2, and ILC3—as well as NK cells. Although NK cells are functionally considered cytotoxic ILC1s, they are usually classified separately because they represent “mature” killer cells, whereas other ILC1s primarily regulate immunity through cytokine secretion (38).

Under pathological conditions, such as follicular lymphoma, ILC populations are markedly perturbed compared with non-malignant tissues (39). ILC1s generally exert anti-tumor activity through IFN-γ production, but sustained activation can drive functional exhaustion (40). ILC2s secrete granzyme B and directly lyse tumor cells via pyroptosis and/or apoptosis, regulated by DNAM-1–CD112/CD155 interactions that inactivate the negative regulator FOXO1 (41). In tumor-bearing mice, pulmonary ILC2s, as well as tumor-infiltrating ILC2s adoptively transferred at a ratio of 1:60 relative to tumor cells, significantly enhance the infiltration of CD4^+^ and CD8^+^ T cells and eosinophils into the tumor microenvironment, thereby suppressing tumor growth. Human ILC2s similarly demonstrate potent anti-tumor activity *in vivo (* 42). ILC3s, on the other hand, interact with T cells through MHC-II, supporting microbial colonization and promoting type 1 immune responses within both the intestine and the tumor (43).

### 2.6 NKT cells

Natural killer T (NKT) cells, a unique subset of T cells, bridge the gap between innate and adaptive immune responses. They are characterized by the expression of invariant T cell receptors (TCRs) and possess the ability to recognize lipid antigens presented by the non-polymorphic MHC-I molecules, specifically CD1d.

Within the TME, however, the expansion of NKT cells is often impaired, leading to a reduced population and an increased rate of cellular senescence (44). Moreover, dysregulated lipid metabolism in the TME alters the composition of lipid antigens presented by CD1d, thereby compromising NKT cell activation and function (45). Despite these inhibitory conditions, NKT cells retain significant anti-tumor potential. Studies have demonstrated that higher infiltration levels of NKT cells are associated with favorable prognoses in several malignancies, including chronic lymphocytic leukemia (CLL) and neuroblastoma (45, 46). In HCC, NKT cells play a crucial role in tumor suppression by secreting IFN-γ (47). Their activation shifts macrophage polarization toward the M1 phenotype while inhibiting M2 macrophages, thereby promoting the clearance of TAMs and suppressing tumor proliferation. In certain tumors, their protective effects are diminished, NKT cells may even facilitate tumor metastasis and progression through the secretion of IL-22 (48).

### 2.7 γδ T cells

T lymphocytes can be classified into αβ T cells and γδ T cells, distinguished by the expression of the αβ TCR and γδ TCR, respectively. γδ T cells are a unique subset of T cells with innate immune characteristics. As rapid responders in the innate immune system, they quickly recognize and eliminate infected or abnormal cells. Unlike conventional T cells, which rely on MHC molecules to present antigens, γδ T cells can directly recognize lipid antigens or stress-induced molecules on the surface of tumor cells, greatly expanding their range of antigen recognition (49).

Based on the TCR chain types (e.g., Vδ1, Vδ2, Vγ4, Vγ6) and their tissue-specific distribution, γδ T cells can be further categorized into subsets with distinct immune functions. For instance, Vδ1^+^ γδ T cells recognize and kill tumor cells via NKG2D ligands; γδT17 cells secrete IL-17, which is correlated with tumor metastasis. Conversely, γδ regulatory T cells (γδTreg) can recruit myeloid-derived suppressor cells (MDSCs), thereby promoting the formation of an immunosuppressive microenvironment (50–52).

### 2.8 B-1 cells

Conventional B cells are a crucial component of the adaptive immune system, primarily generated in the bone marrow, where they play essential roles in antigen recognition, antibody production, and antigen presentation. In contrast, B-1 cells are a subset of B lymphocytes that are mainly involved in innate immunity and are primarily found in the pleural and peritoneal cavities (53). B-1 cells synthesize natural immunoglobulin M (IgM) antibodies and bind to various pathogen-associated carbohydrate antigens, making them pivotal in the initial defense against bacterial infections. In addition to their strong, non-specific response to bacteria and carbohydrate antigens, B-1 cells are also capable of phagocytosing and clearing apoptotic cells (54).

In abdominal tumors, B-1 cells can rapidly produce and secrete natural IgM antibodies that target tumor-associated carbohydrate antigens, thereby promoting tumor cell killing (55). However, the overactivation of B-1 cells is linked to certain diseases, particularly CLL (56). Within the TME, interactions between B-1 cells and melanoma cells enhance the survival of B-1 cells and promote tumor cell metastasis by upregulating the expression of metastasis-associated genes, such as MMP-9 and CXCR-4 (57).

### 2.9 Mast cells

Mast cells (MCs) play a crucial role in immune regulation, performing functions such as secreting various cytokines, expressing MHC molecules, and presenting antigens. They are often significantly increased in both tumor tissues and adjacent areas, and are among the first immune cells recruited to the tumor site, where they participate in tumor initiation and progression (58).

In bladder cancer, mast cells activate interferon and NF-κB signaling pathways, leading to increased secretion of CCL2 and IL-13. This attracts THP-1 monocytes and further promotes tumor progression (59). In contrast, in prostate cancer, mast cells exhibit a negative regulatory role, inhibiting tumor angiogenesis and growth. However, those located in the surrounding tumor microenvironment contribute to tumor cell proliferation (60). In summary, mast cells play a complex and context-dependent role in tumor biology, with their functions varying significantly across different tumor types and microenvironments.

### 2.10 Eosinophils

Eosinophils are a key subpopulation of white blood cells that play diverse roles in immune defense, particularly in parasitic infections, allergic reactions, and immune regulation. In certain solid tumors, elevated eosinophil levels are often associated with favorable prognoses (61, 62). However, in pancreatic cancer, tumor cells can inhibit eosinophil accumulation by secreting chemokines through an autocrine feedback mechanism, thereby facilitating tumor progression (63). Beyond their regulatory roles, eosinophils also contribute to immune activation. For instance, they secrete IL-4, which inhibits CD8^+^ T cell apoptosis and supports the formation of memory CD8^+^ T cells (64). Within the TME, eosinophils activated by TNF-α and IFN-γ release chemokines such as CXCL9 and CXCL10, promoting CD4^+^ and CD8^+^ T cell infiltration and enhancing anti-tumor immune responses (65). Conversely, eosinophils can also facilitate tumor cell migration and metastasis by secreting CCL6 (66). Therefore, eosinophils play a dual and context-dependent role in tumor immunity, capable of both enhancing immune responses and contributing to tumor progression depending on the tumor type and microenvironment.

### 2.11 Basophils

Basophils are a subset of white blood cells found in both blood and tissues, traditionally recognized for their role in allergic responses. However, emerging research highlights their equally important involvement in innate immunity (67). Within the TME, basophils display complex and context-dependent functions, capable of both suppressing and promoting tumor progression. In pancreatic ductal carcinoma, activated basophils have been shown to exert significant tumor-suppressive effects, helping to inhibit further tumor development. Conversely, in other contexts, basophils may support tumor growth by secreting cytokines such as IL-13, which reduce the proportion of Th1-like immune cells and dampen anti-tumor immunity (68). The pro-tumorigenic role of basophils is particularly evident in hematologic malignancies. For instance, in patients with chronic myeloid leukemia (CML), basophils express hepatocyte growth factor (HGF), which promotes the proliferation of CML cells (69). In solid tumors, basophils can influence the immune microenvironment to support tumor development. In lung cancer, for instance, they drive the generation of immunosuppressive myeloid cells through the IL-4 signaling axis, thereby facilitating tumor growth (70).

### 2.12 MDSCs

MDSCs are derived from myeloid cells, which are key components of the innate immune system, their classification as innate immune cells remains controversial. The primary role of innate immune cells is to rapidly identify and eliminate pathogens, whereas MDSCs primarily function to suppress immune responses (71). In healthy individuals, MDSCs are virtually absent and typically expand only under pathological conditions. Thus, despite their myeloid origin and close ties to the innate immune system, MDSCs primarily act as immunosuppressive cells rather than typical innate immune effectors. However, some researchers argue that MDSCs can be considered a “regulatory branch” of the innate immune system due to their role in maintaining immune balance in the inflammatory microenvironment (72).

The recruitment of MDSCs is a critical step in the formation of an immunosuppressive TME, primarily mediated by chemokine receptors CXCR2, CCR2, and CCR5 (73, 74). In the TME, MDSCs not only foster immune suppression through interactions with diverse lymphoid and myeloid cells but also secrete inhibitory cytokines that limit the infiltration and activity of cytotoxic CD8^+^ T cells (75). Additionally, MDSCs produce large amounts of ROS and nitric oxide, both of which inhibit T cell function and further promote tumor progression (76). Moreover, studies have shown that MDSCs and TAMs engage in bidirectional interactions, significantly amplifying the immunosuppressive effect (77).

## 3 Innate immune factors

In the previous section, we discussed the critical roles of innate immune cells in tumor immune surveillance and immune escape. As regulators of these immune cells, innate immune factors play an essential role in quickly identifying and eliminating pathogens, while also serving as a bridge for immune responses. Not only do they provide the foundation for the body’s early defense, but they also facilitate adaptive immunity by delivering crucial signals, forming a comprehensive immune defense network. In this section, we will explore the role of innate immune factors in greater detail.

### 3.1 Complement system

The complement system, composed of over 60 proteins and regulatory factors, represents a complex branch of innate immunity. Its activation can promote tumor clearance through opsonization, phagocytosis, and complement-dependent cytotoxicity (CDC) (79–81). However, aberrant or sustained complement activity within the tumor microenvironment often drives pro-tumorigenic processes, including TAM polarization, angiogenesis, recruitment of immunosuppressive MDSCs, and MMP-9–mediated metastasis, highlighting its dual role in regulating tumor metabolism (82).

Mechanistic studies provide illustrative examples of complement-mediated modulation of TAMs. In ovarian cancer, aberrant C5aR expression on TAMs induces an immunosuppressive phenotype, whereas genetic or pharmacological C5aR blockade reprograms TAMs, restores CXCL9 production, enhances CD8^+^ T cell infiltration, and improves the efficacy of immune checkpoint inhibition (83). In glioma, the NFAT1–C3a–C3aR feedback loop maintains M2-like TAM polarization and promotes mesenchymal transition of tumor stem cells, while C3aR inhibition reverses TAM-mediated immunosuppression and limits tumor growth (84). Conversely, complement-activating immunotherapeutic complexes (CoMiX) targeting HER2-positive tumor cells via the alternative pathway (FHR4-mediated) or the classical pathway (tri-Fc dimer–mediated) significantly enhance C3b/C5b-9 deposition and CDC, effectively suppressing tumor growth even in resistant models (85).

Collectively, these findings position the complement system at the interface of innate and adaptive immunity, emphasizing its context-dependent and multifaceted roles in cancer. They highlight the potential of complement-targeted strategies—ranging from pathway-specific inhibitors to innovative complement-activating immunotherapies—to modulate the tumor microenvironment and enhance antitumor immunity (86, 87). Integrating such approaches with existing immunotherapies, guided by context-specific complement profiling, offers a promising path toward more precise, effective, and personalized cancer treatments.

### 3.2 Cytokines

Cytokines are crucial signaling molecules in innate immunity. They rapidly establish a defense network by regulating immune cell recruitment, activation, and function, while also providing a bridge for the activation of adaptive immunity. As key players in the innate immune response, inflammatory cytokines are mainly produced by immune cells in response to pathogens. These cytokines increase vascular permeability and promote the recruitment of immune cells such as neutrophils and monocytes. Chronic inflammation is strongly associated with tumor initiation and progression. Many inflammatory cytokines, such as IL-17, and IL-23, play significant roles in tumor cell proliferation, immunosuppression, and metastasis within the tumor microenvironment (88, 89). These findings underscore the role of chronic inflammation in the tumor microenvironment as a driving force for tumor immune escape.

Chemokines, a subset of cytokines, regulate the migration of immune cells. They are categorized into four main types: CC, CXC, CX3C, and C. By interacting with various G protein-coupled receptors, chemokines form a complex receptor-ligand network that controls immune cell recruitment, activation, and function (90). Within the tumor microenvironment, both tumor cells and immune cells secrete different chemokines that regulate the recruitment of T cells and regulatory T cells (Tregs), influencing the overall tumor immune response (91). For example, the chemokine CXCL10 promotes the infiltration of T cells into the tumor microenvironment, enhancing anti-tumor immunity, while CCL20 recruits Tregs via the FOXO1/CEBPB/NF-κB signaling pathway, thereby promoting chemotherapy resistance in colorectal cancer (91, 92). In addition to immune cell migration, chemokines also contribute to angiogenesis. Tumors maintain their growth and metastasis by secreting pro-angiogenic factors, which help establish an abnormal vascular network (93, 94).

In conclusion, the complement system and cytokines play complex and multifaceted roles in tumor immunity. While they enhance immune defense, they can also create conditions that promote tumor immune escape.

## 4 Mechanisms of innate immune disorders

Tumors significantly impact the immune system, leading to the progressive attenuation of both innate and adaptive immune responses. The role of innate immune cells and immune factors in tumors has been discussed earlier. In this section, we will delve into the mechanisms underlying innate immune disorders in TME, focusing particularly on the interactions between immune signaling pathways, metabolic disorders, and immune cell senescence.

### 4.1 Innate immune signaling is impaired

Innate immune signaling pathways within the TME are crucial for tumor immune surveillance and the immune response. Alterations in these pathways affect the immunogenicity of tumor cells, the activity and functionality of immune cells, as well as the tumor’s response to immunotherapeutic interventions. Recent studies have identified the involvement of various signaling pathways in tumor innate immunity, notably the cGAS-STING pathway, Toll-like receptors (TLRs), RIG-I-like receptors (RLRs), NOD-like receptors (NLRs), and C-type lectin receptors (CLRs).

The cyclic guanosine monophosphate-adenosine monophosphate synthase (cGAS), a cytoplasmic receptor for double-stranded DNA (dsDNA), promotes the synthesis of the second messenger cGAMP upon activation. cGAMP then interacts with the STING protein to activate downstream signaling pathways, triggering immune responses (95). Notably, type I interferon (I-IFN) plays a key role in detecting tumor cell immunogenicity and activating tumor-specific CD8^+^ T cells, a process that depends on STING-mediated signaling (96). Moreover, STING has been shown to enhance tumor cell survival through the activation of the IFN/STAT1 signaling pathway, underscoring its complex and dual role in tumor immunity (97).

TLRs, as pattern recognition receptors (PRRs), are essential for innate immune responses. Aberrant TLR activation can promote tumorigenesis and immune evasion (98). TLRs recognize specific components of pathogens and initiate signaling cascades, such as those mediated by myeloid differentiation factor 88 (MyD88). Most TLRs activate NF-κB via MyD88-dependent signaling, triggering inflammatory responses and influencing DC maturation (99). In lung cancer, silencing TLR2, TLR4, and TLR9, along with epithelial-specific MyD88/NF-κB signaling, significantly reduces the expression of pro-tumor immunosuppressive factors like RETNLB, while enhancing the expression of anti-tumor cytokines like IFN-γ (100). In breast cancer cells, activation of TLR2 and TLR4 stimulates NF-κB, regulating the secretion of cytokines such as IL-6, TGF-β, VEGF, and MMP9, promoting tumor invasion, migration, and progression (101, 102).

RLRs are key immune receptors that detect viral RNA in the cytoplasm. Upon binding to RNA ligands, RLRs trigger downstream signaling cascades that activate transcription factors like IRF3 and NF-κB, leading to the production of I-IFN and inflammatory cytokines, which are critical for antiviral responses (103). In bladder cancer, the upregulation of the m6A reading factor YTHDF2 inhibits RIG-I, reducing CD8^+^ T cell recruitment to the TME (104). In bladder cancer, RIG-I–mediated type I interferon signaling is suppressed, contributing to immune evasion (105).

NLRs play an important role in the innate immune response. NLRs recognize pathogen-associated molecular patterns (PAMPs) and damage-associated molecular patterns (DAMPs), and participate in immune defense by activating NF-κB and MAPK signaling pathways (106). Studies show that activation of the NOD1 pathway accelerates tumor progression, while NOD2 loss is associated with a protective effect in colitis-related tumors (107, 108). However, another study found an increased tumor incidence in NOD2 knockout mice, suggesting that the role of NOD2 in tumorigenesis may not be related to intestinal microbial imbalance (109).

CLRs are a key class of pattern recognition receptors predominantly expressed by myeloid cells. CLRs have significant roles in both innate and adaptive immune responses by recognizing pathogens and DAMPs released from necrotic cells (110). In gastric cancer, CLR Dectin-1 is associated with poor prognosis and promotes immune evasion by modulating the immunosuppressive activity of TAMs (111). Furthermore, CLR Dectin-2-mediated activation of the Nlrp3 inflammasome enhances NK cell function and inhibits liver metastasis (112). These observations illustrate that CLR signaling intersects with inflammasome pathways to produce divergent effects. CLRs also play a role in tumor glycan recognition and dendritic cell dysfunction, driving immunosuppression and tumor immune escape (113). Such seemingly contradictory outcomes likely reflect differences in the cellular compartments involved (tumor, myeloid, or stromal cells), the temporal dynamics of inflammasome activation, and the influence of organ-specific microenvironments.

### 4.2 Metabolic disorders

In the TME, the metabolic profile of innate immune cells plays a pivotal role in regulating their function. Glycolysis and lipid metabolism are two key pathways involved in immune cell activation and response. Disruptions in these metabolic processes not only impair immune cell efficacy but also contribute to tumor growth, metastasis, and immune evasion.

**Figure 2:**
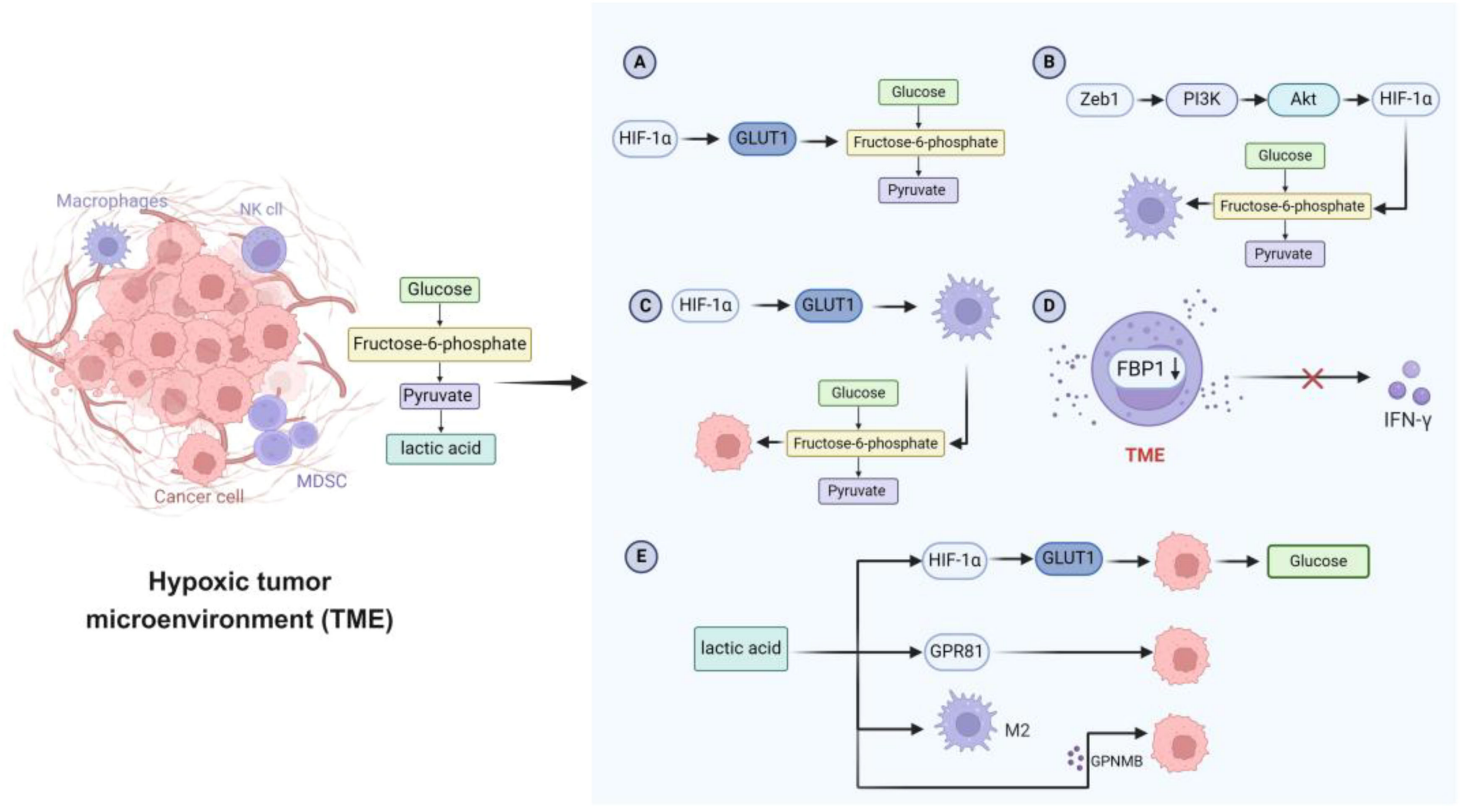
Characteristics of glucose metabolism in hypoxic TME. **(A)** Tumor cells activate HIF-1α, thereby increasing the expression of GLUT and promoting the glycolytic flux of tumor cells. **(B)** The transcription factor Zeb1 enhances glycolytic activity through the PI3K/Akt/HIF-1α signaling pathway and promotes the transformation of macrophages to a preneoplastic phenotype. **(C)** GLUT1 is involved in enhancing macrophage glycolysis and supporting tumor cell growth. **(D)** Abnormal expression of FBP1 in NK cells in TME inhibits glycolysis, impairs NK cell viability, and limits IFN-γ secretion. **(E)** Lactate enhances glucose uptake in tumor cells by up-regulating the expression of GLUT1. Lactate also affects tumor cell growth and metastasis through GPR81 receptor. In addition, lactate induces the polarization of macrophages to an M2-like phenotype and regulates the secretion of GPNMB, which further promotes the migration and invasion of tumor cells. TME, tumor microenvironment; HIF-1α, hypoxia-inducible factor 1α; GLUT, glucose transporter; Zeb1, zinc finger E-box binding homeobox 1; PI3K, phosphatidylinositol 3-kinase; Akt, protein kinase B; FBP1, fructose-1,6-bisphosphatase 1; NK, natural killer; IFN-γ, interferon-γ; GPR81, G-protein coupled receptor 81; GPNMB, glycoprotein non-metastatic melanoma protein B.

Glycolysis provides a rapid source of energy for innate immune cells following activation. Key glycolytic enzymes such as hexokinase (HK), phosphofructokinase (PFK), pyruvate kinase (PK), and lactate dehydrogenase (LDH) are upregulated in this process (**Figure 2**) (114). Meanwhile, the rapid proliferation of tumor cells and angiogenesis create a hypoxic TME, which activates HIF-1α. HIF-1α further enhances the expression of glycolytic enzymes and glucose transporters (e.g., GLUT1), increasing glycolytic flux to meet the energy demands of both tumor and immune cells (115). Innate immune cells in the TME also display distinct glucose metabolism patterns. For instance, TAMs show enhanced glycolysis, which supports their immunosuppressive phenotype. In breast cancer, the transcription factor ZEB1 promotes macrophage glycolysis via the PI3K/Akt/HIF-1α pathway, facilitating their shift to a pro-tumor state and reinforcing immunosuppression within the TME (116). Moreover, upregulation of GLUT1 further supports tumor progression. In contrast, NK cells in the TME exhibit downregulation of glycolysis-related genes. Elevated expression of fructose-1,6-bisphosphatase (FBP1), which inhibits glycolysis, weakens NK cell activity and reduces IFN-γ secretion (117, 118). Lactate—the end product of glycolysis—accumulates in the TME and acts as a key metabolic signal, disrupting CD8^+^ T cell activation and antitumor immunity by interfering with GLUT10-mediated glucose transport (119).

Beyond glycolysis, lipid metabolism is equally critical in innate immune regulation. Fatty acid oxidation (FAO), a key lipid metabolic pathway, supplies significant energy to tumor cells. Enzymes involved in FAO, such as fatty acid synthase (FASN), sterol regulatory element-binding protein 1 (SREBP1), and carnitine palmitoyltransferase 1 (CPT1), are commonly upregulated in various tumors, supporting their rapid growth (120, 121). Under hypoxic conditions, HIF-1α inhibits FAO by suppressing acyl-CoA dehydrogenase, thereby promoting tumor survival (122). Pyruvate, a glycolytic intermediate, may either be converted into lactate or enter the mitochondria to fuel fatty acid synthesis (123). Lipid accumulation within the TME also influences immune cell function. Oxidized lipids taken up by DCs form covalent complexes with heat shock protein 70 (HSP70), impairing MHC-I translocation to the cell membrane and disrupting antigen presentation (124). Similarly, CD8^+^ T cells lose their cytotoxic function after excessive lipid uptake via CD36, due to lipid peroxidation and activation of the p38 signaling pathway (125).

Cholesterol homeostasis within immune cells is regulated mainly by SREBP2, which promotes cholesterol synthesis and uptake, and liver X receptor (LXR), which drives cholesterol efflux (126). Tumor cells exploit these pathways to accumulate cholesterol, supporting their continuous proliferation (127, 128). Interestingly, cholesterol levels vary across cell types in the TME: tumor-infiltrating lymphocytes (TILs) and TAMs often exhibit cholesterol deficiency, whereas tumor cells and bone marrow-derived immunosuppressive cells maintain elevated cholesterol levels (126, 129). Cholesterol depletion, particularly in CD8^+^ cytotoxic T cells, has a notable impact on their anti-tumor capacity. Several tumor-derived factors also influence cholesterol metabolism in the TME. For example, APOA1 from glioblastoma, and CSF1 from prostate cancer can promote cholesterol efflux from TAMs (129, 130). The released cholesterol is then reabsorbed by prostate cancer cells and used for dihydrotestosterone synthesis, which activates androgen receptor signaling and downstream gene expression—ultimately accelerating tumor progression (131, 132). Nevertheless, the broader regulatory effects of cholesterol efflux from innate immune cells on other TME components remain to be fully understood.

### 4.3 Cellular senescence

In the TME, cellular senescence is increasingly recognized not only as a stress response of tumor cells but also as a key regulator of immune function. Tumor cells are subjected to a variety of chronic stressors—such as oncogenic signals, replication stress, hypoxia, ROS, nutrient deprivation, and inflammatory factors—which can drive them into a state of senescence. Although senescent cells lose their proliferative capacity, they remain metabolically active and influence the surrounding microenvironment through the senescence-associated secretory phenotype (SASP). This secretory profile enables senescent cells to exert strong paracrine effects, particularly on immune cell behavior. For instance, DCs co-cultured with senescent tumor cells show enhanced antigen-presenting capabilities, suggesting that SASP factors can promote antigen presentation under specific conditions (133). The TME can also facilitate the senescence of immune cells. Tumor stem cells release the IL-6, a pro-aging factor that induces macrophage senescenc (134). Furthermore, tumor cells and Tregs can influence lipid metabolism and induce T cells senescence by upregulating the expression of phospholipase A2, contributing to tumor immune evasion (135, 136).

## 5 Targeted innate immunotherapy

**Figure 3:**
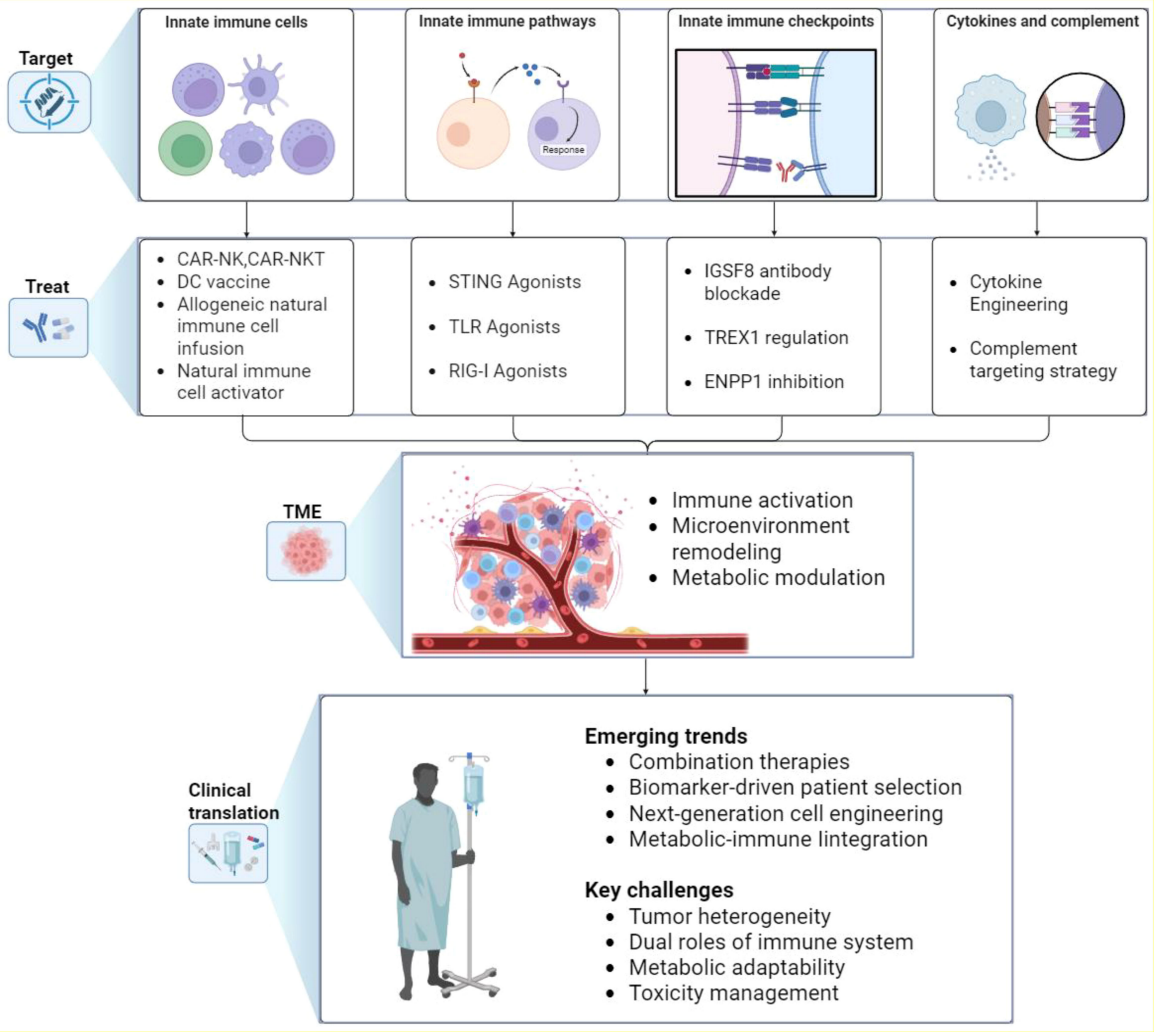
Therapeutic strategies targeting innate immunity in cancer. The figure summarizes current and emerging approaches to harness innate immunity in cancer therapy. Four major target areas are illustrated: innate immune cells, innate immune pathways, innate immune checkpoints, and innate immune factors such as cytokines and complement. Corresponding therapeutic strategies aim to reprogram innate immune responses, which in turn modulate the TME by enhancing immune activation, remodeling tissue context, and reshaping metabolic activity. These effects are being translated into clinical applications, with key emerging trends including rational combination therapies, biomarker-driven patient selection, next-generation cell engineering, and metabolic–immune integration. Major challenges that remain include tumor heterogeneity, the dual roles of innate immune mediators, metabolic adaptability, and toxicity management. TME, tumor microenvironment; TAM, tumor-associated macrophage; NK, natural killer; DC, dendritic cell; ILC, innate lymphoid cell; CAR, chimeric antigen receptor; TLR, Toll-like receptor; STING, stimulator of interferon genes.

Innate immunity plays a critical role in tumor initiation and progression, and accumulating evidence from both basic research and clinical practice indicates that its therapeutic modulation can substantially enhance anti-tumor efficacy. To provide a comprehensive overview of this rapidly evolving field, we summarize the main therapeutic targets—including innate immune cells, innate signaling pathways, innate checkpoints, and innate immune factors such as cytokines and complement—along with their impact on the TME and key opportunities and challenges for clinical translation (**Figure 3**). Complementing this conceptual summary, representative clinical trials are highlighted to illustrate the progress and limitations of current strategies (**Table 2**). The following subsections expand on these approaches in detail.

**Table 2:** Clinical studies targeting innate immunity.

Type of target	Drugs	Route of administration	Tumors	Clinical trials	Phase
CD7+T cell	CAR7 T	Intravenous injection	Acute T lymphoblastic leukemia	ISRCTN15323014 (137)	1
CD3+T cell	glofitamab	Intravenous injection	B-NHL	NCT03075696 (138)	1
NK cell	CAR-NK	Intravenous injection	Non-hodgkin’s lymphoma, CLL	NCT03056339 (141)	2
NK cell	mbIL-21	Intravenous injection	Acute myeloid leukemia	NCT01787474 (142)	1
DC	DCVax-L	Subcutaneous Administration	Glioblastoma multiforme	NCT00045968 (144)	3
DC	DCVAC/PCa	Subcutaneous Administration	Prostate Cancer	NCT 01520051 (143)	3
γδ T cell	Allogeneic Vγ9Vδ2 T cells	Intravenous injection	Acute myeloid leukopenia	NCT03790072 (140)	1
Macrophages	Bexmarilimab	Intravenous injection	HCC, biliary tract cancer, colorectal cancer, ovarian cancer, pancreatic ductal adenocarcinoma, melanoma, gastric adenocarcinoma, breast cancer, and anaplastic thyroid cancer	NCT03733990 (145)	2
NKT cell	Anti-GD2 CAR-NKT	Intravenous injection	Neuroblastoma	NCT03294954 (146)	1
cGAS-STING	Manganese	Intravenous injection	Multiple solid tumors	NCT03991559 (149)	1
TLR9	1018 ISS	Subcutaneous Administration	Follicular lymphoma	NCT00251394 (150)	2
TLR9	GNKG168	Intravenous injection	AL	NCT01743807 (151)	1
RLR	MK-4621	Intratumoral administration	Lymphoma	NCT03065023 NCT03739138 (152)	1 1
CD19+T cell	huCART19-IL18	Intravenous injection	lymphoma	NCT04684563 (159)	1
complement factor H	GT103	Intravenous injection	Non-small cell lung cancer	NCT04314089 (160)	1b

### 5.1 Targeting innate immune cells

Targeting innate immune cells represents a novel immunotherapeutic approach aimed at enhancing or reactivating their ability to identify and eliminate tumor cells. Among these, chimeric antigen receptor T cell (CAR-T) therapy remains one of the most extensively studied modalities. Several CD19-targeting CAR-T therapies have already been approved for clinical use, demonstrating promising therapeutic outcomes. Notably, Robert Chiesa’s team is developing a CD7-targeted CAR-T therapy, which has shown preliminary efficacy in specific tumor types (137). Additionally, Glofitamab, a bispecific antibody targeting CD3 on T cells, has achieved sustained complete remission in patients with refractory and aggressive B-cell non-Hodgkin’s lymphoma (B-NHL), further expanding the landscape of immunotherapy (138). Merging strategies targeting γδ T cells have also shown broad therapeutic potential across various tumor types (139). γδ T cells derived from haploidentical donors have demonstrated promising safety and efficacy in a phase I clinical trial involving patients with refractory or relapsed acute myeloid leukemia (140).

Beyond CAR-T cells, chimeric antigen receptor-modified natural killer cells (CAR-NK) have attracted growing interest. By introducing CARs into NK cells, their tumor recognition and cytotoxic capabilities are significantly enhanced. Clinical trials have reported that CAR-NK therapy improves response rates in CD19^+^ malignancies and prolongs both overall survival (OS) and progression-free survival (PFS) (141). Moreover, NK cells derived from patients or healthy donors can be expanded ex vivo and reinfused, offering a feasible and scalable therapeutic option (142).

DC vaccines also hold considerable promise in cancer immunotherapy. For instance. Early-phase studies demonstrated immunogenicity and potential survival benefit, but outcomes in larger randomized trials have been inconsistent—for example, DCVAC/PCa in mCRPC showed no OS advantage, whereas DCVax-L in glioblastoma reported encouraging signals but raised concerns about trial design and patient selection (143, 144).

Macrophage-targeted therapy has also emerged as a promising avenue. Bexmarilimab, an inhibitor of CLEVER-1-mediated macrophage activation, has demonstrated early signs of improving survival in patients with various solid tumors (145). Furthermore, chimeric antigen receptor natural killer T cell (CAR-NKT) therapy, an innovative approach, showed good safety and preliminary efficacy in a first-in-human clinical trial, achieving an objective response rate of 25% (3 out of 12 patients) (146).

### 5.2 Targeting innate immune pathways

Activation of innate immune pathways plays a crucial role in initiating antitumor immune responses. In recent years, agonists TLRs and the STING pathway have shown promising potential in clinical trials. Although clinical application of the cGAS-STING pathway is still in its early stages, several studies have reported encouraging safety profiles and preliminary efficacy in immune activation (147, 148). For instance, research by Lv Mengze’s team demonstrated that the synergistic use of manganese ions and PD-1 antibodies activates the cGAS-STING signaling pathway, thereby promoting the infiltration and maturation of CD8^+^ T cells, DCs, and macrophages, ultimately enhancing antitumor immunity (149).

TLR agonists have also gained attention for their potent immunomodulatory properties. The TLR-9 agonist 1018 ISS, when combined with rituximab, significantly increased CD8^+^ T cell and macrophage infiltration in tumor tissues (150). Another TLR-9 agonist, GNKG168, was shown to independently reduce NK cell immunosuppression in patients with acute leukemia (151). Additionally, the RIG-I agonist MK-4621 exhibited notable antitumor activity both as a monotherapy and in combination with PD-1 blockade (152). Currently, multiple innate immune pathway agonists are undergoing clinical evaluation, and the results are eagerly anticipated.

### 5.3 Targeting innate immune checkpoint

Immune checkpoint inhibitors (ICIs) have profoundly transformed the landscape of tumor immunotherapy by unleashing adaptive immune responses, particularly through blockade of classical targets such as PD-1 and CTLA-4. However, accumulating evidence suggests that the innate immune system also harbors functionally significant checkpoint molecules, thereby representing promising candidates for next-generation immunotherapeutic strategies (153). Among these, IGSF8 has emerged as a novel inhibitory checkpoint, capable of suppressing NK cell activity via its interaction with human KIR3DL2 or murine Klra9 receptors. Preclinical studies have demonstrated that antibody-mediated blockade of this pathway can restore NK cell cytotoxicity against malignant cells *in vitro*, highlighting its potential therapeutic value (154). In addition to surface checkpoint receptors, intracellular regulators such as the DNA exonuclease TREX1 have been implicated in shaping antitumor immunity. The loss of TREX1 in tumor cells can initiate robust activation of both CD8^+^ T cells and NK cells, mitigate T cell exhaustion, and reprogram the immunosuppressive myeloid microenvironment, collectively enhancing the efficacy of immunotherapy (155). Another promising target is ectonucleotide pyrophosphatase/phosphodiesterase 1 (ENPP1), frequently overexpressed in a variety of malignancies and closely associated with the formation of an immunosuppressive tumor microenvironment (156).

### 5.4 Targeting cytokines and complement

In addition to innate immune cells, signaling pathways, and checkpoint molecules, innate immune factors such as cytokines and the complement system are increasingly recognized as actionable targets in cancer immunotherapy.

Cytokines have long been studied for their potent immunomodulatory activity. Early agents such as IFNα and high-dose IL-2 were approved for selected malignancies but were limited by modest efficacy and significant toxicities (157). Building on these milestones, next-generation cytokine therapeutics are being developed, including engineered “superkines,” fusion proteins with extended half-lives and tumor-targeted activity, and antagonists of immunosuppressive cytokines (158). An innovative approach is armored CAR-T cells engineered to secrete IL-18 (huCART19-IL18), which demonstrated robust expansion, acceptable safety, and durable clinical responses in relapsed/refractory lymphoma (159). These advances underscore how cytokine engineering can enhance both the efficacy and persistence of cell-based therapies.

The complement system, another ancient arm of innate immunity, exerts dual roles in cancer progression: while capable of mediating opsonization and cytotoxicity, persistent activation via the C5a/C5aR1 axis fosters TAM recruitment, immune suppression, and metastasis (86). Complement regulators such as CD46, CD55, CD59, and factor H are frequently upregulated in tumors, contributing to immune evasion (87). Clinical translation is now underway, exemplified by the first-in-class anti–factor H antibody GT103, which showed safety and disease stabilization in a phase 1b trial of refractory non-small cell lung cancer (160). Complement-targeted strategies are also being tested in combination with checkpoint blockade to reprogram the tumor microenvironment and promote effector T cell infiltration.

Together, cytokine- and complement-based therapies expand the therapeutic armamentarium of innate immunotherapy. By integrating these innate immune factors–targeting approaches with established cell- and checkpoint-directed strategies, next-generation immunotherapies may achieve more durable and personalized clinical benefit.

## 6 Challenges, controversies, and future directions

Despite major progress in dissecting the role of innate immunity in tumor development and therapy, many unresolved questions and conflicting findings remain. These challenges span innate immune cells, innate immune factors, and metabolic regulation, each presenting both opportunities and uncertainties for clinical translation.

### 6.1 Innate immune cells

Innate immune cells often display paradoxical, context-dependent roles. For example, subsets of γδ T cells exert potent cytotoxicity, whereas γδT17 or γδTreg cells can drive angiogenesis and immunosuppression. Dendritic cell vaccines have demonstrated immunogenicity in early studies, yet randomized clinical trials have produced inconsistent benefits. TAMs also exhibit duality, with some subsets enhancing antigen presentation while others facilitate immune evasion. Similarly, NK cells show strong efficacy in hematologic malignancies but limited persistence and activity in solid tumors, partly due to inhibitory checkpoint interactions. These examples highlight the high heterogeneity of innate immune cells and underscore the need for standardized definitions, patient stratification based on immune subsets, and reprogramming strategies rather than indiscriminate expansion or depletion.

### 6.2 Innate immune factors

Innate immune mediators, including complement and cytokines, likewise play dual roles. Complement can drive cytotoxicity and opsonization, yet sustained C3a/C5a signaling recruits immunosuppressive myeloid cells and supports angiogenesis. Cytokine therapies illustrate a similar paradox: while early agents such as IFNα and IL-2 validated the concept, they were limited by toxicity and variable efficacy. Next-generation cytokines, such as engineered superkines and fusion proteins, offer improved activity but remain highly dependent on tumor microenvironmental context. Progress will therefore require predictive biomarkers of efficacy and toxicity, along with rational combinations with checkpoint inhibitors or cell-based therapies.

### 6.3 Metabolic pathways

Metabolic reprogramming and innate immune signaling are closely intertwined, together shaping either pro- or anti-tumor responses. The accumulation of metabolites such as lactate, oxidized lipids, and altered cholesterol flux reshapes bone marrow and lymphoid function, promoting immunosuppressive TAM and MDSC phenotypes while impairing T and NK cell activity. Dysregulation of innate pathways, exemplified by contradictory reports on NOD2, further illustrates this complexity: depending on context, such signaling may enhance immune surveillance or drive tumor progression through chronic inflammation and microbiota-related dysbiosis.

These intertwined mechanisms provide therapeutic opportunities but also significant barriers. Although metabolic interventions—such as glycolysis inhibitors, LDH inhibitors, FAO/FASN modulators, and agents targeting cholesterol metabolism—can restore immune activity in preclinical models, their translation is hindered by systemic toxicity, compensatory metabolic rewiring, and tumor-type–specific effects. Overcoming these obstacles will require tumor-targeted delivery systems (e.g., nanoparticles, tumor-activated prodrugs) and biomarker-guided patient selection.

In summary, whether at the level of innate immune cells, immune factors, or metabolism, cancer immunity is characterized by profound context dependence and frequent contradictions. Addressing these challenges will require standardized models, integration of single-cell and spatial multi-omics, and metabolic flux tracing to establish causal links. Ultimately, biomarker-driven clinical trials and rational combinations of metabolic modulators with immunotherapies—such as checkpoint blockade, adoptive cell transfer, or engineered cytokines—will be essential to safely and effectively harness innate immunity as a foundation for durable and personalized cancer treatment.

## 7 Conclusion

This review explores the complex interplay between innate immunity and cancer. Innate immunity plays a critical role in anti-tumor responses by recognizing and eliminating tumor cells and contributing to immune surveillance. However, tumor cells employ various immune evasion mechanisms that weaken immune recognition, thereby promoting tumor growth and metastasis. Within the TME, metabolic disturbances further suppress innate immune function, enhance immunosuppression, and facilitate immune escape, accelerating cancer progression. A comprehensive understanding of the mechanisms underlying the interaction between innate immunity and tumor cells is therefore essential for developing more effective immunotherapeutic strategies.

Despite progress in this field, significant challenges remain. For instance, no universal tumor-associated patterns have been identified that are consistently recognized by the innate immune system. While innate immunity can detect certain tumor-related molecular or cellular changes, these changes are not exclusive to tumors and may also occur in non-cancerous conditions such as tissue injury or metabolic stress, limiting specificity and selectivity. Enhancing the tumor recognition and cytotoxic capabilities of the innate immune system has thus become a key objective in improving cancer control. Additionally, the safety and long-term efficacy of therapies targeting innate immunity require further investigation.

Future research should aim to better characterize the immune and metabolic landscape of the TME and elucidate the precise role of innate immunity in anti-tumor defense. Exploring combination therapies that harness the full potential of innate immunity alongside other treatment modalities may offer promising new avenues for cancer treatment.
